# SARS-CoV-2 infection in farmed minks, the Netherlands, April and May 2020

**DOI:** 10.2807/1560-7917.ES.2020.25.23.2001005

**Published:** 2020-06-11

**Authors:** Nadia Oreshkova, Robert Jan Molenaar, Sandra Vreman, Frank Harders, Bas B Oude Munnink, Renate W Hakze-van der Honing, Nora Gerhards, Paulien Tolsma, Ruth Bouwstra, Reina S Sikkema, Mirriam GJ Tacken, Myrna MT de Rooij, Eefke Weesendorp, Marc Y Engelsma, Christianne JM Bruschke, Lidwien AM Smit, Marion Koopmans, Wim HM van der Poel, Arjan Stegeman

**Affiliations:** 1 Wageningen Bioveterinary Research, Wageningen University and Research, Lelystad, the Netherlands; 2 GD Animal Health, Deventer, the Netherlands; 3 Department of Viroscience, Erasmus University Medical Center, Rotterdam, the Netherlands; 4 Regional Public Health Service Brabant-Zuid-Oost, Eindhoven, the Netherlands; 5 Institute for Risk Assessment Sciences (IRAS), Utrecht University, Utrecht, the Netherlands; 6 Ministry of Agriculture, Nature and Food Quality, The Hague, the Netherlands; 7 Department of Farm Animal Health, Faculty of Veterinary Medicine, Utrecht University, the Netherlands

**Keywords:** SARS-CoV-2, mink, interstitial pneumonia, transmission

## Abstract

Respiratory disease and increased mortality occurred in minks on two farms in the Netherlands, with interstitial pneumonia and SARS-CoV-2 RNA in organ and swab samples. On both farms, at least one worker had coronavirus disease-associated symptoms before the outbreak. Variations in mink-derived viral genomes showed between-mink transmission and no infection link between the farms. Inhalable dust contained viral RNA, indicating possible exposure of workers. One worker is assumed to have attracted the virus from mink.

Currently, humanity is facing a pandemic of a new coronavirus, severe acute respiratory syndrome coronavirus 2 (SARS-CoV-2). The virus is spreading efficiently among people, causing predominantly respiratory disease with varying degree of severity. The virus has also been shown to infect a number of animal species under experimental conditions. Rhesus and cynomolgus macaques, ferrets, cats and golden Syrian hamsters supported viral replication in respiratory tract and some of those species (rhesus macaques, juvenile cats and hamsters) displayed a mild to moderate clinical disease [1-9]. Besides the experimental infections, occasional spillover from humans to domestic or captive animals has been reported. In a few isolated cases, cats and dogs owned by infected individuals tested positive for SARS-CoV-2 RNA and occasionally, cats also displayed clinical disease [10,11]. Recently, several tigers in the Bronx zoo (New York City, United States (US)) with respiratory symptoms were confirmed positive for SARS-CoV-2 [10]. In all cases, a direct correlation with infected humans was established or at least other sources of infection were excluded [10].

Here, we report SARS-CoV-2 infection of minks on two farms in the Netherlands and describe the associated clinical signs, pathological and virological findings. Sequence analysis of mink-derived viruses pointed at humans as the probable source of the initial infection and demonstrated transmission between minks. Furthermore, the presence of viral RNA in inhalable dust collected from the farms indicated a possible exposure of workers to virus excreted by minks.

## Mink farming background

Minks are farmed for their fur. In the Netherlands, there are around 125 mink farms, with an average of 5,000 female breeding animals. In 2019, 4 million minks were produced. The sector has around 1,200 full-time and 400 part-time employees [12]. On two mink farms (NB1 and NB2) situated in the south of the Netherlands, province North Brabant (NB), an increased mink mortality was observed mid-April 2020, which coincided with display of respiratory signs in some animals. On NB1, 13,700 animals are housed in two separate, but closely situated houses (house A and house B, 115 m apart), which are served by the same personnel and vehicles. NB2 has 7,500 animals. Farms NB1 and NB2 are 14 km apart from each other. There was no connection of workers, vehicles or animal transports, between these two farms. On both farms, minks are individually housed in wire netting cages with a nest box. The cages are arranged in long single rows, separated by feeding alleys. The two cage sides that border other cages are solid, made of wood or plastic, ensuring that there is no direct animal-to-animal contact. The cage rows are situated inside halls, which provide a roof, but are largely open to the wind from the sides. Both farms are family-owned and besides the four (NB1) and two (NB2) members of the farmer family, one and six employees were working on the farms, respectively.

## Disease history and clinical observations

**Figure 1:**
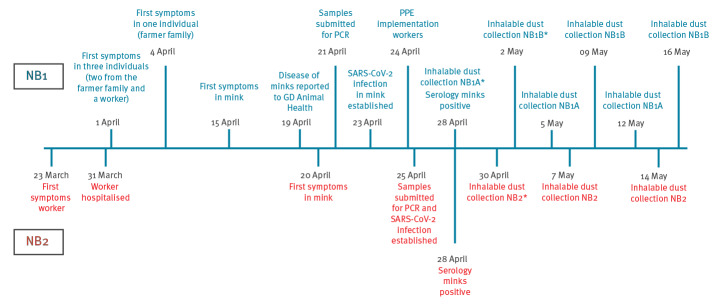
Schematic representation of the time-line of events in the first month of a SARS-CoV-2 outbreak on two mink farms, the Netherlands, April 2020

Covid-19: coronavirus disease; NB1/NB2: Farms 1 and 2 in North Brabant; PPE: personal protective equipment; SARS-CoV-2: severe acute respiratory syndrome coronavirus 2.

Signs of respiratory disease in the animals were reported on 19 and 20 April 2020 (Figure 1) on NB1 and NB2, respectively. The symptoms were mostly limited to watery nasal discharge, but some animals showed severe respiratory distress. The exact numbers of animals that displayed symptoms, as well as the severity of the symptoms, were not registered. On both farms, the veterinarian was consulted when severe respiratory disease symptoms were observed by the farmer. Animals that had died were necropsied and tested for SARS-CoV-2, influenza A, adenoviral infection, *Escherichia coli* and *Pseudomonas aeruginosa*. All tests except SARS-CoV-2 were negative. Overall mortality between date of reporting and 30 April was 2.4% at NB1 and 1.2% at NB2, while ca 0.6% would have been expected, based on observations from previous years, in the same period. Affected animals were not concentrated in a specific location, but rather scattered throughout the buildings of each farm. At this time of the year, the mink populations consist mainly of pregnant females. In the few litters that were already present, no increase in pup mortality was noticed.

Dates of significant events are shown with the corresponding findings. Cases of (suspected) human Covid-19 infections in farm workers that coincided with the SARS-CoV-2 infection of the minks are excluded from this report. Analysis of those cases is currently being performed and will be reported in a subsequent publication. Asterisks indicate the dates on which dust samples collected inside the mink farm buildings were found positive for viral RNA.

Lungs from three recently died animals per farm were collected and submitted for qPCR analysis on 21 (NB1) and 25 (NB2) April. One sample per farm was also sequenced (index samples). In the following week, 36 recently dead animals were collected (18 per farm) and necropsied. A throat and rectal swab were taken from each animal for qPCR analysis.

## Pathological analysis

### Macroscopic findings 

**Figure 2:**
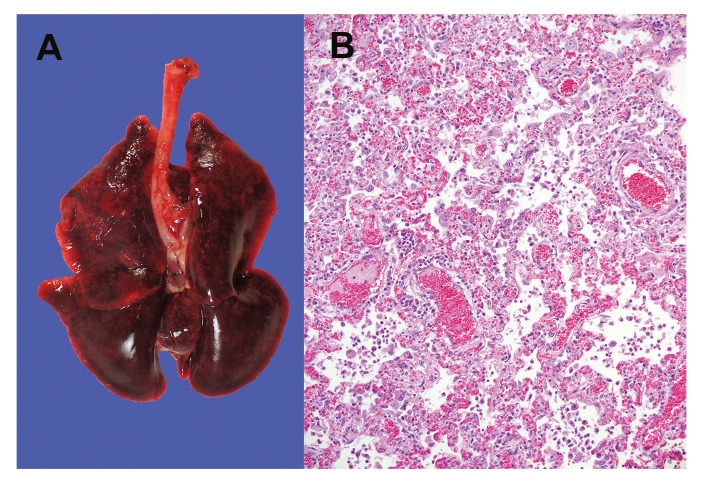
Lung from a necropsied mink, SARS-CoV-2 outbreak on two mink farms, the Netherlands, April 2020

The necropsies revealed that 16 of 18 animals from NB1 and 12 of 18 from NB2 had diffusely dark to mottled red, wet lung lobes that did not collapse when opening the thoracic cavity, indicating interstitial pneumonia (Table 1 and Figure 2A). Other investigated organs displayed no significant macroscopic changes. Minks without the described lung findings had macroscopic changes consistent with either chronic Aleutian disease, septicaemia, or dystocia. From seven animals with clear macroscopic lung changes, organs were harvested for histopathological and virological investigation.

**Table 1:** Gross pathology and cause of death of necropsied minks, SARS-CoV-2 outbreak on two mink farms, the Netherlands, April 2020 (n = 36)

Farm NB1				Farm NB2			
**Animal number**	**Date of death^a^**	**Date of necropsy**	**Cause of death**	**Animal number**	**Date of death^a^**	**Date of necropsy**	**Cause of death**
1	28 Apr	28 Apr	Interstitial pneumonia	1	27 Apr	27 Apr	Sepsis and lung oedema with congestion
2^b^	28 Apr	28 Apr	Interstitial pneumonia	2^b^	27 Apr	27 Apr	Interstitial pneumonia
3	28 Apr	28 Apr	Interstitial pneumonia	3	27 Apr	27 Apr	Aleutian disease
4	28 Apr	28 Apr	Interstitial pneumonia	4	27 Apr	27 Apr	Aleutian disease
5	28 Apr	28 Apr	Interstitial pneumonia	5	27 Apr	27 Apr	Sepsis
6	28 Apr	28 Apr	Interstitial pneumonia	6	27 Apr	27 Apr	Dystocia
7	28 Apr	28 Apr	Interstitial pneumonia	7^b^	27 Apr	27 Apr	Interstitial pneumonia
8	28 Apr	28 Apr	Interstitial pneumonia	8^b^	27 Apr	27 Apr	Interstitial pneumonia
9	28 Apr	28 Apr	Aleutian disease	9	26 Apr	27 Apr	Interstitial pneumonia
10	28 Apr	28 Apr	Interstitial pneumonia	10	26 Apr	27 Apr	Interstitial pneumonia
11	28 Apr	28 Apr	Interstitial pneumonia	11	26 Apr	27 Apr	Interstitial pneumonia
12	28 Apr	28 Apr	Interstitial pneumonia	12	26 Apr	27 Apr	Interstitial pneumonia
13^b^	28 Apr	28 Apr	Interstitial pneumonia	13	26 Apr	27 Apr	Interstitial pneumonia
14^b^	28 Apr	28 Apr	Interstitial pneumonia	14	26 Apr	27 Apr	Interstitial pneumonia
15	28 Apr	28 Apr	Interstitial pneumonia	15	26 Apr	27 Apr	Interstitial pneumonia
16^b^	28 Apr	28 Apr	Interstitial pneumonia	16	26 Apr	27 Apr	Interstitial pneumonia
17	28 Apr	28 Apr	Interstitial pneumonia	17	26 Apr	27 Apr	Interstitial pneumonia
18	28 Apr	28 Apr	Interstitial pneumonia	18	26 Apr	27 Apr	Sepsis

SARS-CoV-2: severe acute respiratory syndrome coronavirus 2.

^a^ Date when the animals were found dead (animals are inspected daily).

^b^ Organs from those animals were collected for SARS-CoV-2 qPCR.

SARS-CoV-2: severe acute respiratory syndrome coronavirus 2.

Panel A: Representative macroscopic image of an affected lung. Panel B: Representative microscopic image (objective 20×) of a section of the lung, fixed in 10% formalin and stained with haematoxylin and eosin, showing interstitial pneumonia.

### Histological findings 

A severe diffuse interstitial pneumonia with hyperaemia, alveolar damage and loss of air containing alveolar lumina was detected in all the seven harvested lungs (Figure 2B). Bacterial cultures from the organs of the seven animals were negative.

## Virus detection and sequencing

Presence of viral RNA was determined by qPCR against the SARS-CoV-2 E gene (Table 2) [13]. Viral RNA was detected in the conchae, lung, throat swab and rectal swab of all seven minks from which organs were collected. In addition, viral RNA was detected in the liver of one, and in the intestines of three animals. Spleens of all seven animals were negative for viral RNA (Table 2). In the swabs collected from all 36 necropsied animals, viral RNA was detected in all throat swabs and 34 of the 36 rectal swabs. The cycle threshold (Ct) values varied, but were on average lower in the throat swabs than in the rectal swabs (average Ct = 21.7 and 31.2, respectively), indicating higher viral loads in the throat swabs.

**Table 2:** Virus titres, determined by qPCR in organs and swabs of necropsied minks, SARS-CoV-2 outbreak on two mink farms, the Netherlands, April 2020 (n = 36)

	Animal number	Conchae	Lung	Spleen	Liver	Distal large intestines	Throat swab	Rectal swab
Farm NB1	2	8,25	4,54	Not detected	Not detected	4,22	6,87	3,30
	13	9,16	5,17	Not detected	Not detected	3,56	6,81	3,01
	14	8,08	3,83	Not detected	Not detected	Not detected	7,04	3,95
	16	7,08	3,90	Not detected	Not detected	4,97	6,47	4,47
Farm NB2	2	8,19	5,77	Not detected	Not detected	Not detected	8,03	2,58
	8	8,55	5,55	Not detected	3,45	Not detected	7,30	3,84
	7	8,46	5,98	Not detected	Not detected	Not detected	6,69	5,42

SARS-CoV-2: severe acute respiratory syndrome coronavirus 2.

Titres were calculated based on a calibration curve of a virus stock with a known infectious virus titre and are expressed as log_10_ (median tissue culture infectious dose (TCID50)/g of tissue (organ material) or log_10_ TCID50/mL of swab material (swabs were always submerged in 2 mL of cell culture medium).

**Figure 3:**
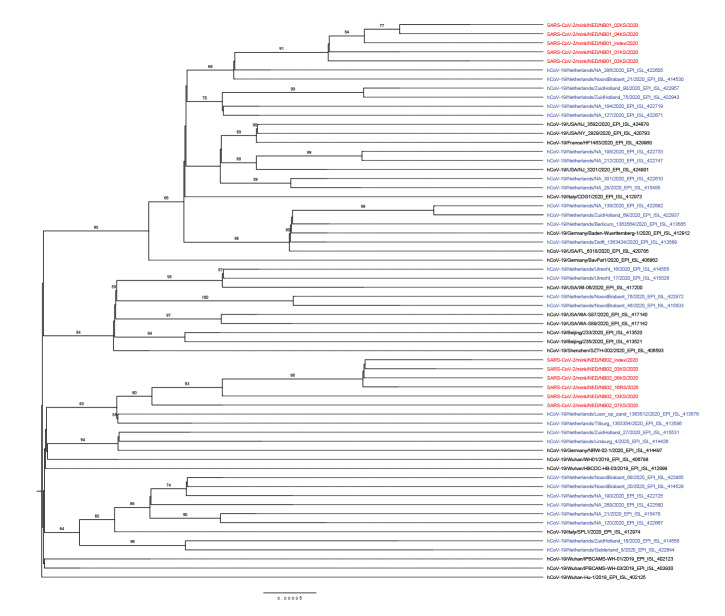
Maximum likelihood phylogenetic tree of SARS-CoV-2 sequences from minks and selected full-length sequences from the GISAID EpiCoV database

GISAID: Global Initiative on Sharing All Influenza Data; SARS-CoV-2: severe acute respiratory syndrome coronavirus 2.

The viral sequences of the index samples and from additional four and five animals from NB1 and NB2, respectively, were determined by next generation sequencing and deposited in GenBank (MT396266 and MT457390–MT457399). Phylogenetic analysis of the sequences suggests separate virus introductions to each of the farms (Figure 3). The index sequences show nine (NB1) and 15 (NB2) nucleotide substitutions across the complete genome in comparison with Wuhan-Hu-1 (NC_045512.2, EPI_ISL_402125). The two index sequences diverge at 22 nucleotide positions, but the sequences from each farm cluster together (Figure 3). Mink-specific single nucleotide polymorphisms were found in ORF1a, ORF1b, spike, ORF3, ORF7a and 3’UTR (Supplementary Table 2).

Red: sequences from minks described in this report; blue: Dutch sequences from GISAID; black: international sequences from GISAID.

Details on the sequences from GISAID (https://www.gisaid.org/) used for this analysis, based on proposed lineages [14], are listed and acknowledged in Supplementary Table 1). The collected sequences were aligned using MAFFT v7.427 [15] and the evolutionary history was inferred by RAxML version 8.2.12 [16] using the maximum likelihood method based on the general time reversible model with a gamma-distributed variation of rates and 1,000 bootstrap replicates. The tree is rooted at Wuhan-Hu-1. Bootstrap support values above 50 are indicated at the corresponding branch.

## History of coronavirus disease in farm workers

Farm owners and their families were interviewed by the public health service for possible history of disease. Four persons on farm NB1 have had respiratory disease symptoms compatible with Covid-19 since beginning of April, including three members of the farmer’s family and a worker (Figure 1). These people were not investigated for SARS-CoV-2 infection. At NB2, one worker had been diagnosed with SARS-CoV-2 infection and hospitalised on 31 March (Figure 1). A clinical sample was retrieved, but the viral load was too low for sequencing analysis. At farm NB1, one person who stayed on the farm, showed mild respiratory disease and was diagnosed with SARS-CoV by 28 April. Based on preliminary sequencing results, this person was assumed to have attracted the virus from mink. A further detailed investigation focusing on the transmission of the virus between humans and mink on the farms is ongoing.

## Sampling of the environment and stray cats

Inhalable dust samples were collected three times between 28 April and 16 May (Figure 1) by active stationary air sampling during 5–6 h, using Gilian GilAir 5 pumps (Sensidyne, St. Petersburg, US) at 3.5 L/min, total dust sampling system (Gesamtstaubprobenahme; GSP) sampling heads (JS Holdings, Stevenage, United Kingdom) and Teflon filters (Pall Corporation, Ann Arbor, US). In each mink house, sampling was conducted at three different locations. Viral RNA was detected in two of the three samples from NB1, house A (Ct = 35.95 and 38.18) and in one of three samples from NB1, house B (Ct = 35.03) and from NB2 (Ct = 35.14) on the first sampling moment, but all samples were negative on the second and third sampling moments.

A total of 24 stray cats found in the surroundings of the farms NB1 and NB2 were sampled for SARS-CoV-2 infection by collecting serum and oropharyngeal swabs. Seven cats had antibodies against SARS-CoV-2, detected by an in-house virus microneutralisation assay, and one cat was positive for viral RNA. However, the amounts of viral RNA were very small, and we were unable to generate a sequence from this cat. The sampled stray cats inhabit the surroundings of the farms, but do not come into the houses of people.

## Discussion

Here we present a report of infection of two mink farms with SARS-CoV-2. While this manuscript was being prepared, similar SARS-CoV-2 outbreaks occurred on another nine farms in the Netherlands, eight in the province Noord Brabant and one in the province Limburg. On farms NB1 and NB2 described here, coronavirus disease (COVID-19)-like symptoms were present in people working on the farms before signs were seen in the minks, and SARS-CoV-2 infection was confirmed in one hospitalised person. The viral sequences obtained from the mink samples were closely related to sequences of human-derived isolates. The distance between the two sequence clusters originating from the two farms suggests separate introductions, arguing against an epidemiological connection between the two farms. Whether the outbreaks on the rest of the farms were connected to the first two cases and between each other is being investigated. The most likely explanation for the widespread infection on the mink farms is introduction of the virus by humans and subsequent transmission among the minks. Ferrets, which are closely related to minks, were also able to transmit the virus to other ferrets under experimental conditions; transmission was observed under both direct and indirect contact (animals were housed in cages with a permeable partition separating infected from uninfected animals) [5]. Minks can be housed in cages with permeable separation between them, which could have explained animal-to-animal transmission. On the mink farms in question however, animals are caged separately with non-permeable partition between cages, precluding direct contact as a mode of transmission. Indirect transmission between minks could either be through fomites (e.g. by feed or bedding material provided by humans), by infectious droplets generated by the infected animals, or by (faecally) contaminated dust from the bedding.

Detection of viral RNA in the airborne inhalable dust on the mink farms clearly suggests dust and/or droplets as means of transmission between the minks and occupational risk of exposure for the workers on the farms. While the exact occupational hazard for humans is currently being determined, to anticipate the exposure risk for personnel working on the mink farms with confirmed SARS-CoV-2 infections, the public health authorities in the Netherlands have issued an advice for all workers on infected mink farms to wear personal protective equipment including face masks, goggles, gloves and overalls, while fulfilling their work duties [17]. Visitors are prohibited to enter those farms. Mink farm workers who have COVID-19 symptoms are advised to stay at home. Mandatory screening of all Dutch mink farms was started on 28 May and is aimed to be completed by 15 June. On 3 June, the Dutch Ministry of Agriculture decided to cull all minks of SARS-CoV2 infected farms, starting on 5 June [18].

Mink farms are present in other countries in Europe, China and the US but so far, SARS-CoV-2 infections in these animals have been reported only in the Netherlands. The purpose of the current report is to raise awareness in the scientific community and in the mink industry that minks are susceptible for SARS-CoV-2. Infected animals developed respiratory disease with typical pathological findings of viral pneumonia and were able to transmit the virus among each other. While this manuscript was in preparation, also serological surveillance was performed on the farms NB1 and NB2. Sixty random serum samples were collected from the minks of each farm and were all found positive for SARS-CoV-2 neutralising antibodies, except one sample from NB1. These findings coincided with the disappearance of symptoms and mortality on the farms and were followed by inability to detect viral RNA in inhalable dust, suggesting that the SARS-CoV-2 outbreaks were widely spread within the farms and resolved on their own when the majority of animals had seroconverted. There are still a lot of questions to address, especially regarding possible transmission from mink to human and exposure risks for the public outside the farms. In this report, we showed that humans can become a source of infection for minks, which results in a disease outbreak. Human infections acquired from mink are also suspected and data on exposure risk for humans as well as samples of potentially Covid-19-infected people on the farms are being collected and analysed; forthcoming results will be published in the future.

## Supplementary Data

491000 Supplement
