# Correction for Euro Surveill. 2020;25(23)

**DOI:** 10.2807/1560-7917.ES.2021.26.12.210325c

**Published:** 2021-03-25

**Authors:** Eurosurveillance editorial team

**Affiliations:** 1 European Centre for Disease Prevention and Control (ECDC), Stockholm, Sweden

The supplement accompanying the article ‘SARS-CoV-2 infection in farmed minks, the Netherlands, April and May 2020’ by Oreshkova et al., published on 11 June 2020, was replaced on 25 March 2021 on request of the authors. The update corrects the amino acid numbering of mutations in Supplementary Table 2.

